# The association between social deprivation and the prevalence and severity of dental caries and fluorosis in populations with and without water fluoridation

**DOI:** 10.1186/1471-2458-12-1122

**Published:** 2012-12-28

**Authors:** Michael G McGrady, Roger P Ellwood, Anne Maguire, Michaela Goodwin, Nicola Boothman, Iain A Pretty

**Affiliations:** 1 School of Dentistry, University of Manchester, Manchester, M13 9PL, UK; 2 Colgate Palmolive Dental Health Unit, Williams House, Lloyd Street North, Manchester, M15 6SE, UK; 3 School of Dental Sciences, University of Newcastle, Newcastle, UK

## Abstract

**Background:**

To determine the association between social deprivation and the prevalence of caries (including caries lesions restricted to enamel) and enamel fluorosis in areas that are served by either fluoridated or non-fluoridated drinking water using clinical scoring, remote blinded, photographic scoring for caries and fluorosis. The study also aimed to explore the use of remote, blinded methodologies to minimize the effect of examiner bias.

**Methods:**

Subjects were male and female lifetime residents aged 11–13 years. Clinical assessments of caries and fluorosis were performed on permanent teeth using ICDAS and blind scoring of standardized photographs of maxillary central incisors using TF Index (with cases for fluorosis defined as TF > 0).

**Results:**

Data from 1783 subjects were available (910 Newcastle, 873 Manchester). Levels of material deprivation (Index of Multiple Deprivation) were comparable for both populations (Newcastle mean 35.22, range 2.77-78.85; Manchester mean 37.04, range 1.84-84.02). Subjects in the fluoridated population had significantly less caries experience than the non-fluoridated population when assessed by clinical scores or photographic scores across all quintiles of deprivation for white spot lesions: Newcastle mean DMFT 2.94 (clinical); 2.51 (photo), Manchester mean DMFT 4.48 (clinical); 3.44 (photo) and caries into dentine (Newcastle Mean DMFT 0.65 (clinical); 0.58 (photo), Manchester mean DMFT 1.07 (clinical); 0.98 (photo). The only exception being for the least deprived quintile for caries into dentine where there were no significant differences between the cities: Newcastle mean DMFT 0.38 (clinical); 0.36 (photo), Manchester mean DMFT 0.45 (clinical); 0.39 (photo). The odds ratio for white spot caries experience (or worse) in Manchester was 1.9 relative to Newcastle. The odds ratio for caries into dentine in Manchester was 1.8 relative to Newcastle. The odds ratio for developing fluorosis in Newcastle was 3.3 relative to Manchester.

**Conclusions:**

Water fluoridation appears to reduce the social class gradient between deprivation and caries experience when considering caries into dentine. However, this was associated with an increased risk of developing mild fluorosis. The use of intra-oral cameras and remote scoring of photographs for caries demonstrated good potential for blinded scoring.

## Background

In the second half of the 20^th^ Century the fluoridation of community water supplies was introduced in several countries around the world in order to address the high prevalence of dental caries. Such arrangements were implemented following expansive research by H Trendley Dean in the United States [1-3]. In the United Kingdom during the 1950’s, following observations in the United States, several pilot water fluoridation arrangements were introduced in order to evaluate water fluoridation as a public health measure. Ultimately, the only major UK localities still receiving fluoridated community water supplies are the West Midlands and Newcastle upon Tyne.

There have been numerous studies evaluating the use of water fluoridation. In the Netherlands, a major longitudinal study investigated the effects of fluoridating the water supply of Tiel and comparing the patterns of caries prevalence and severity with non-fluoridated Culemborg. The study ran from 1953 until 1971 and found differences between the localities in caries severity with significantly fewer white spot lesions in Tiel progressing into cavitated lesions compared to non-fluoridated Culemborg. In Tiel 93% of buccal and 86% interproximal lesions had not progressed into dentine compared to 65% buccal and 65% interproximal lesions in Culemborg [4-7].

Similar studies in the UK have demonstrated reductions in caries in populations following the introduction of water fluoridation [8,9]. Studies conducted in Newcastle and non-fluoridated Northumberland demonstrated similar differences in caries levels between the two populations when compared to studies conducted elsewhere in the UK [10-15]. When fluoridation arrangements have ended, as in the case of Anglesey where capital investment for new equipment was deemed economically unviable, it has been demonstrated that caries levels increase following cessation of fluoridation [16].

As the use of fluoridated dentifrices became increasingly popular during the 1970’s and 1980’s, the differences in caries rates between fluoridated and non-fluoridated populations were reduced. Caries prevalence declined in both fluoridated and non-fluoridated populations and whilst there were still significant differences between caries rates in fluoridated and non-fluoridated populations, the differences were no longer as great as they had been during the 1950’s and 1960’s. In addition to this, there had been an increase in the prevalence of mostly mild fluorosis [17]. Furthermore, owing to confounding factors such as halo effects and identifying sources of fluoride, it has become more difficult to investigate the impact of water fluoridation over and above the use of fluoridated dentifrice alone [18-20].

The link between social deprivation and ill health has been known for many years [21,22]. This is also reflected in oral health where despite overall reductions in caries levels there are still persistent inequalities between the social classes [23]. Studies conducted in the UK have shown differences in child caries levels between areas of high and low deprivation including comparisons between fluoridated and non-fluoridated populations suggesting water fluoridation may reduce inequalities in health relating to dental caries by reducing the social gradient [24-32].

There are several means of measuring deprivation within a population and data are generally reported as summary measures to assist in the exploration of other dependent variables. Two commonly used indices in dental research are Townsend’s Index of Material Deprivation [33] and the Jarman Deprivation Score [34]. More recently, the Index of Multiple Deprivation (IMD) has become popular as a means of reporting deprivation at a Local Super Output Area (LSOA) geographical level [35]. The IMD has seven domains with indicators in each domain that are measured separately. The seven domains are: income, employment, health, education (skills and training), barriers to housing, crime and living environment. A weighting of these seven domains provides an overall area level aggregate score.

The York Report [36] concluded “the evidence of a benefit of a reduction in caries should be considered together with the increased prevalence of dental fluorosis.” Certain aspects within the evidence base highlighted the need to improve the quality of research. Consideration should be given to increases in prevalence of dental fluorosis where evidence showed a benefit of a reduction in dental caries. The report also stated the evidence base required improvement relating to potential harm or the impact on social inequalities. Another report followed in the UK from the Medical Research Council (MRC) that echoed the views of the York report relating to the need to improve the evidence base [37]. The MRC report recommended appropriate measures of social inequalities were needed for research focused on water fluoridation, dental caries and fluorosis.

Despite the fact overall population caries levels have declined in recent decades, there remains a problem in certain demographic groups, which include young children and those with high levels of social deprivation. As the dental profession moves towards a preventive approach rather than restorative, the need for more sensitive methods of detection has increased. In the UK the British Association for the Study of Community Dentistry (BASCD) has conducted a series of national surveys relating to dental health (now known as the NHS Dental Epidemiology Programme (DEP)). Traditionally the survey has employed the use of the DMF index using trained examiners following criteria defined for the age group in question. The “D” or decayed component employed by BASCD uses a diagnostic threshold of visual caries into dentine (D_3_). Whilst this has been a useful means for screening and surveillance it is now questionable if assessing caries at this gross threshold will be acceptable in the future when assessing the impact of preventive measures dependent on the need for early detection in order to increase the likelihood of preventing caries or reversing early enamel lesions [38-41]. There is a need to develop methods of detecting and monitoring early carious lesions with high levels of validity and reliability.

The International Caries Detection and Assessment System (ICDAS) was developed to define visual caries detection criteria at an early non-cavitated stage that could inform diagnosis, prognosis and clinical management [42,43]. The ability of the ICDAS system to enable detection of early, non-cavitated (white spot) lesions provides an opportunity to explore caries prevalence in fluoridated and non-fluoridated populations to determine if there are differences between these populations at low levels of caries severity as well as the more established assessment of caries at a diagnostic threshold of caries into dentine. This will permit possible comparisons with data generated from the Tiel-Culemborg study in the Netherlands with respect to the progression of white spot lesions in to cavitated lesions and possible effects of water fluoridation on the prevalence and severity of caries.

The aims of this study were to determine the association between social deprivation and the prevalence of caries (including caries lesions restricted to enamel) and enamel fluorosis (on the maxillary central incisors) in areas served by either fluoridated or non-fluoridated drinking water. The study also aimed to explore the use of remote, blinded methodologies to minimize the effect of examiner bias using clinical scoring and remote blinded photographic scoring employing ICDAS criteria for caries and TF Index for fluorosis.

## Methods

The study was conducted in two localities with and without fluoridated community water supplies, Newcastle upon Tyne (Fluoridated at 1 ppm F) and Greater Manchester (non-fluoridated). Ethical approval for the study was obtained from the University of Manchester Committee on the Ethics of Research on Human Beings (ref: 07952). Permission was sought from relevant Local Authorities to approach schools in their locality. Schools were selected based upon the percentage free school meals entitlement (%FSME) to provide a spectrum of socio-economic backgrounds [44] and their willingness to participate. Letters were sent to the parents of male and female pupils in years 7 and 8 (aged 11–13) containing information sheets for parents and pupils together with parental opt-out forms with a stamped addressed envelope to return to the study team if the parent or carer did not wish their child to participate. Two weeks before the scheduled school visit a reminder and further opportunity to opt-out was sent to each parent who had not previously returned an opt-out form.

The study ran between February 2008 and December 2009. Blocks of examination time were arranged to take into consideration school availability during term time and were balanced between the localities to minimize examination bias and to ensure the age ranges of the subjects were comparable between the localities.

### Screening and selection of participants

Pupils whose parents had not returned an opt-out form attended for recruitment and were invited to participate in the study. Written informed consent was obtained for each subject. During the recruitment phase, lifetime residency in the locality and residential postcode were confirmed. Subjects who were not lifetime residents were excluded from the study. Postcode details for each participant enabled an individual level measure of social deprivation to be ascribed using the Index of Multiple Deprivation (IMD) by linking the postcode with the LSOA IMD score. Consented subjects were asked to complete a short pictorial computer based questionnaire on oral hygiene practices: type of brush, quantity of paste and rinsing habits.

### Clinical examinations and image capture

Clinical examinations were undertaken by a single trained examiner (MGM) for caries using ICDAS criteria [42,43] under standard lighting conditions together with a portable chair, air compressor and disposable instruments for examination. Subjects were excluded if they did not possess both maxillary permanent central incisors. Intra-oral images were taken of the teeth using a SOPRO 717 intra-oral camera (Acteon Group, USA) and a bespoke software package written by Dental Health Unit staff (AT) that enabled image capture for each tooth linking it to the subject identifier. The images were integrated into a graphical user interface that randomized and blinded the images that were then displayed on a 32-inch flat screen monitor under controlled lighting (the images produced were viewed at 10x magnification). This ensured the examiner (MGM) was unaware of the area of residence of the subject and each image was scored under identical conditions. This enabled comparison with the clinical caries scores. A selection of subjects from each locality was asked to return for reproducibility scores a minimum of 30 minutes after their initial examination. This was based on logistical and time constraints and subject willingness to return to for examination.

Following the clinical examination, the maxillary central incisors were dried for 1 minute with cotton roll and standardized digital photographs taken using a Nikon D100 camera, Micro Nikkor 105 mm f2.8 lens and a Nikon SB21 ring flash [45]. None of the images contained identifying subject features. The images were exported to a computer and linked to a photographic log using a unique subject identifier. All images were scored remotely by the examiner (MGM) in a blind manner for fluorosis using Thylstrup and Fejerskov (TF) index [46] on completion of the clinical phase of the study using the same methodology as the intra oral images for caries scoring (the images for fluorosis scoring were viewed at 5x magnification). The highest TF score given to either maxillary central incisor was the value recorded for a subject. No substitutions were permitted in the event of missing or un-assessable teeth. A random selection of images was selected in order to obtain reproducibility scores.

The difference in image magnification between the caries and fluorosis scoring was as a result of the different reproduction ratios of the cameras used. The intra-oral camera produced images with a higher degree of magnification compared to the photographic technique employed for fluorosis scoring.

Before completing the study visit, subjects were given instructions on how to complete a 3-day food diary, which would be taken home and completed over 3-days (including one day over the weekend). A random cohort of subjects across both localities were asked to return with their diaries for an in depth interview with a dietician to assess intake of non-milk extrinsic sugars (NMES).

### Data Management and analysis

Data from the caries examinations was recorded on case report forms and entered into Statistical Package for Social Sciences (SPSS 16.0) for statistical analysis. Data for the intra-oral caries images and the fluorosis scores from the photographic images were recorded directly by an interface into a Windows (Microsoft Corp., Seattle, Wash., USA) excel file and imported into SPSS for statistical analysis.

During clinical ICDAS examinations for caries the teeth were thoroughly air dried. In order to take “wet images” of teeth during the photographic examination, the teeth would need to rehydrate with saliva. The period of time required for rehydration would vary upon the size and severity of enamel lesion. Therefore, for logistical reasons and to avoid issues with re-hydration of lesions, only images of dry teeth were taken with the intra-oral camera. To facilitate comparison between clinical and photographic caries scores ICDAS codes 1 and 2 were collapsed and reported as code 2. Caries data for DMFT were calculated for each subject using the ICDAS code for the D component. Caries experience at white spot lesion (or worse) was calculated as D_1-6_MFT (as some ICDAS code 1 lesions would be classified as ICDAS code 2). Caries experience thresholded at visible caries into dentine was calculated as D_4-6_MFT. Surfaces with sealants were considered to be sound.

Demographical, oral hygiene practices and deprivation data were explored using t-tests and Mann–Whitney-U tests (where appropriate) to determine if significant differences existed between the two localities.

Reproducibility measures for clinical caries scores and fluorosis scores were analyzed using the Kappa statistic. Differences between the fluoridated and non-fluoridated localities for proportions of subjects with fluorosis and caries DMFT scores were tested for statistical significance using the chi-square test.

The relative effects of independent variables for age at examination and IMD score on the presence or absence of caries and fluorosis were determined using a logistic regression model for the fluoridated and non-fluoridated localities.

## Results

**Figure 1:**
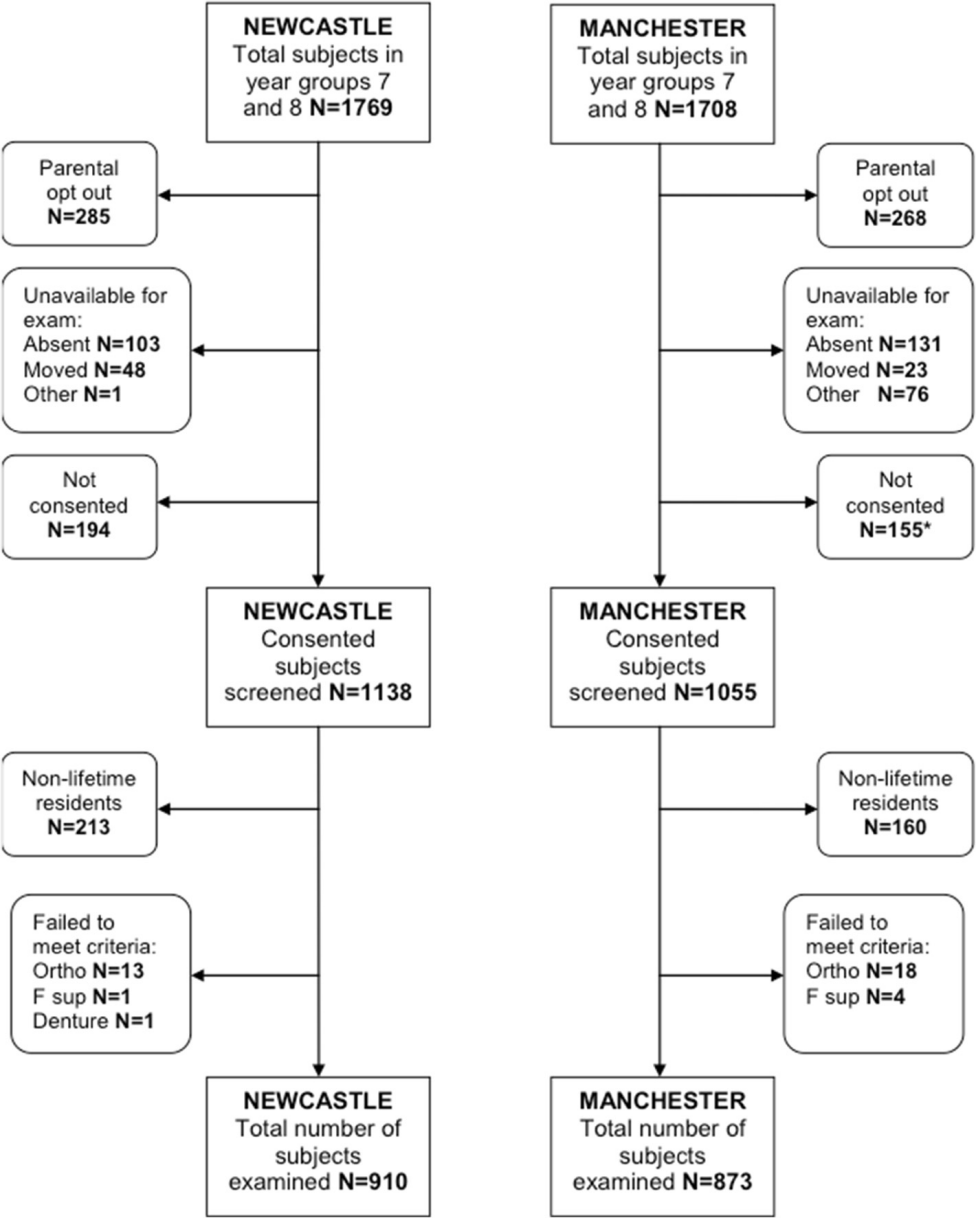
**Subject flow chart.** * includes 2 subjects unable to provide consent.

In total data for 1783 examined subjects were available for analysis. The consent rate when considering all subjects available for examination was 63.1% (64.3% Newcastle; 61.7% Manchester). Of those who consented, 79.9% were examined in Newcastle and 82.7% in Manchester. A flow chart of subjects is shown in Figure 1. Subject demographical data are detailed in Table 1. Overall, measures taken at recruitment to obtain balance between the two localities with respect to age at exam, gender and level of deprivation were generally successful with no significant differences between Newcastle and Manchester.

**Table 1:** Subject demographics

**City**	**Subject numbers**	**Mean age at exam (SD)**	**Gender %**		Mean IMD* (range)
			**M**	**F**	
Newcastle	910	12.56 (0.48)	54	46	35.22 (2.77-78.85)
Manchester	873	12.32 (0.64)	57	43	37.04 (1.84-84.02)
Total	1783	12.44 (0.57)	56	44	36.11 (1.84-84.02)

* IMD – Index of Multiple Deprivation. A compound measure of social deprivation reported by geographical area.

The data in Table 2 summarizes some of the findings from the oral hygiene practices questionnaire and the cohort of subjects that completed the dietary interview (63 Manchester; 65 Newcastle). The cohort data suggested that between the two study areas there were no significant differences either in terms of frequency of NMES intake or NMES consumed in the last hour before bedtime. The oral hygiene practices data revealed no significant differences between the two populations with the exception of rinsing habits where 16% of subjects in Manchester reported not rinsing after brushing compared to only 9% in Newcastle (p = 0.0001). In both populations approximately 40% of subjects reported rinsing with a glass or beaker.

**Table 2:** Summary data for dietary interviews on sugar consumption and oral hygiene practises

	**Manchester (N = 63)**	**Newcastle (N = 65)**	**Significance**
**Diet data - Mean**			
			*Mann Whitney U*
NME^+^ Sugar between meals	1.95	1.86	U = 1989.5, z = −0.284, p = 0.776
NME Sugar last hour before bed	0.48	0.45	U = 2019, z = −0.159, p = 0.874
**Brushing data- Percentiles**			
	**Manchester (N = 873)**	Newcastle (N = 891*)	
**Toothpaste**			
small pea	10%	3%	U =369047, z = −1.954, p = 0.051
thin smear	34%	40%	
large pea	28%	27%	
full brush head	28%	30%	
**Rinse behaviour –**			*Chi square*
No Rinsing	16%	9%	x(2) = 15.9, p = 0.0001**
Wet brush	12%	14%	p = 0.203
Head under tap	19%	18%	p = 0.839
Cupped hands	14%	17%	p = 0.039
Glass or beaker	41%	42%	p = 0.644

*19 subjects had incomplete data and were excluded from examination.

**Significant result.

+NME – non-milk extrinsic sugar.

DMFT data generated from the clinical ICDAS examination for each subject for D_1-6_MFT and D_4-6_MFT are illustrated in Tables 3 and 4. At both thresholds, clinical and photographic DMFT scores for Newcastle were significantly lower than for subjects residing in Manchester (p < 0.0001). The mean D_1-6_MFT in Newcastle was 2.94 (clinical); 2.51 (photo) and for Manchester 4.48 (clinical); 3.44 (photo). For visible caries into dentine the mean D_4-6_MFT in Newcastle was 0.65 (clinical); 0.58 (photo) and for Manchester the mean D_4-6_MFT was 1.07 (clinical); 0.98 (photo). This is illustrated in Table 3.

**Table 3:** Descriptive DMFT data for both cities, at a threshold of white spot lesion and caries into dentine

	**Mean D**_**1-6**_**MFT white spot (SD)**	**95**% **confidence interval**		**Mean D**_**4-6**_**MFT dentine caries (SD)**	**95**% **confidence interval**		**% D**_**1-6**_**MFT >0 white spot**	**% D**_**4-6**_**MFT >0 dentine caries**	**Mean D**_**1-6**_**MFT >0 white spot (SD)**	**Mean D**_**4-6**_**MFT >0 dentine caries (SD)**
		**Lower**	**Upper**		**Lower**	**Upper**				
Manchester (Clinical)	4.48 (3.80)	4.23	4.73	1.07 (1.53)	0.97	1.17	85%	46%	5.29 (3.57)	2.33 (1.47)
Newcastle (Clinical)	2.94 (2.85)	2.76	3.13	0.65 (1.18)	0.58	0.73	75%	32%	3.93 (2.65)	2.01 (1.24)
Manchester (Photo)	3.44 (3.31)	3.22	3.66	0.98 (1.42)	0.88	1.07	80%	46%	4.32 (3.16)	2.15 (1.38)
Newcastle (Photo)	2.51 (2.83)	2.33	2.70	0.58 (1.09)	0.51	0.65	67%	31%	3.74 (2.71)	1.87 (1.19)
2008 12 yr old NHS DEP survey				**Mean D**_**3**_**MFT**	**95% confidence Interval**			**% D**_**3**_**MFT > 0**		**Mean D**_**3**_**MFT > 0**
					**Lower**	**Upper**				
Manchester NHS DEP data				1.12	0.96	1.28		47%		2.36
Newcastle NHS DEP data				0.82	0.72	0.91		38%		2.14

Data from NHS DEP survey 2008 on 12 year olds shown for both cities.

Footnote. The data presented for the DEP is for illustration only – no statistical analyses were conducted.

**Table 4:** Frequency counts for subject DMFT status and comparison between cities for both clinical and photographic scores

	**City**			
	**Clinical**		**Photo**	
	**Newcastle Fluoridated (910)**	**Manchester Non-fluoridated (873)**	**Newcastle Fluoridated (910)**	**Manchester Non-fluoridated (873)**
**Caries D**_**1-6**_**MFT (white spot lesion)**				
**0**	228 (25%)	133 (15%)	298 (33%)	177 (20%)
**1**	115 (13%)	78 (9%)	136 (15%)	134 (15%)
**2**	115 (13%)	92 (10%)	120 (13%)	112 (13%)
**3**	88 (10%)	103 (12%)	87 (10%)	95 (11%)
**4**	169 (19%)	132 (15%)	90 (10%)	91 (10%)
**5**	67 (7%)	53 (6%)	49 (5%)	63 (7%)
**6+**	128 (14%)	283 (32%)	130 (14%)	201 (23%)
	**Mann Whitney U**		**Mann Whitney U**	
	U = 303698, z = −8.683, p < 0.0001		U = 326578, z = −6.950, p < 0.0001	
**Caries D**_**4-6**_**MFT (caries into dentine)**				
**0**	614 (68%)	473 (54%)	626 (69%)	475 (54%)
**1**	134 (15%)	149 (17%)	144 (16%)	165 (19%)
**2**	86 (10%)	111 (13%)	77 (9%)	112 (13%)
**3**	37 (4%)	58 (7%)	36 (4%)	59 (7%)
**4**	25 (3%)	52 (6%)	18 (2%)	44 (5%)
**5**	10 (1%)	18 (2%)	6 (1%)	8 (1%)
**6+**	3 (0.4%)	4 (1%)	3 (0.3%)	10 (1%)
	**Mann Whitney U**		**Mann Whitney U**	
	U = 337110, z = −6.300, p < 0.0001		U = 333436, z = −6.741, p < 0.0001	

**Figure 2:**
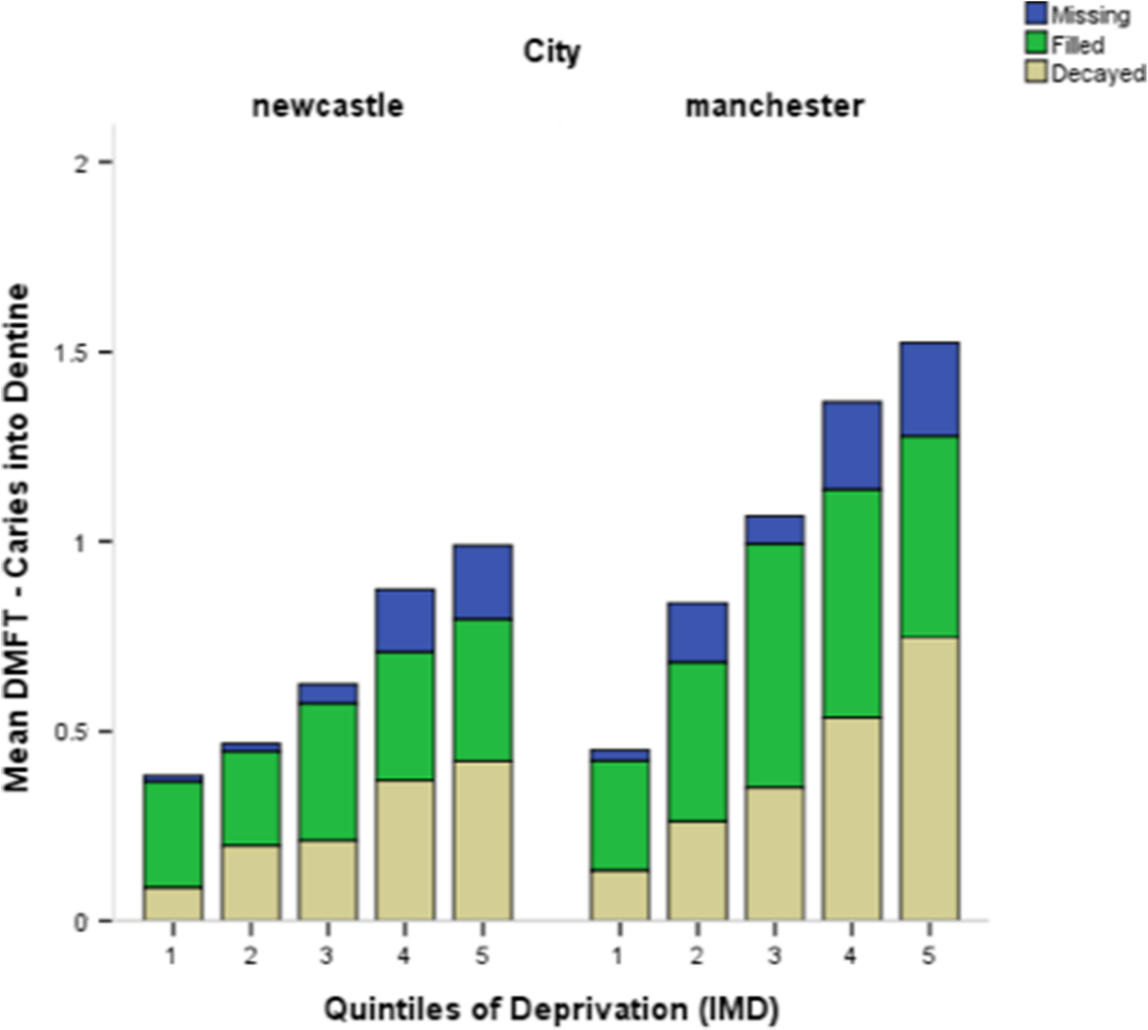
Components of DMFT over each quintile of deprivation depicted for each city.

The percentage of children caries free differed between the two cities for both thresholds of caries detection. In Newcastle 25% were caries free at white spot lesion threshold and 67% for caries into dentine. In Manchester these figures were lower with 15% and 54% respectively for clinical scores (p < 0.0001). Summary data from the NHS DEP 12 year survey for each locality is also shown in Table 3 for illustrative purposes only. The NHS DEP survey was carried out in the same populations during the same time period this study took place. This means the populations in the NHS DEP survey and this study were likely to be similar, although caution should be taken when drawing any conclusions from visual comparisons between the datasets. The components of DMFT for each detection threshold are illustrated in Figure 2 demonstrating the differences between the fluoridated and non-fluoridated populations.

The descriptive data was explored to identify differences between the two localities. Table 4 outlines the frequency distributions between the study groups for DMFT counts for both clinical and photographic scores. The data suggests when detection criteria are set at level of caries into dentine there are clear differences between the fluoridated and non-fluoridated populations (p < 0.0001). However, if the detection threshold is changed to white spot lesion level these differences are reduced but still significant (p < 0.0001). The data sets were comparable between the two scoring techniques, particularly at a threshold of caries into dentine with both techniques (clinical and photographic scoring) demonstrating significant differences between fluoridated Newcastle and non-fluoridated Manchester (p < 0.0001). Data from repeat clinical caries examinations were available for 47 subjects. Weighted Kappa statistics for comparison of ICDAS tooth surface scores were generated at a surface level and showed excellent agreement (weighted Kappa = 0.80) [47]. A similar comparison on 50 subjects was made for ICDAS photographic scores and produced good agreement (weighted Kappa = 0.74).

Comparisons were made between the DMFT scores derived from clinical ICDAS scores and those generated from remote blind scoring of the intra-oral photographs (Tables 3 and 4). Unexpectedly, the photographic DMFT scores were consistently lower than the clinical scores. However, the differences between the two localities were consistent and it was inferred there was minimal effect of bias in the clinical scoring. The data also suggested there appears to be no loss of discrimination using the remote photographic scoring technique i.e. using remote scoring of intra-oral images still separated the fluoridated and non-fluoridated populations.

To explore possible explanations for the lower scores from the photographs crosstab data for ICDAS scores was analyzed between the clinical and photographic techniques. An example is demonstrated in Table 5 illustrating the comparison between scoring techniques for the occlusal surface of the upper right first molar. (Any ICDAS score of 1 was incorporated into ICDAS code 2). It is clear from the data in Table 5 there are some differences in scores between the two techniques particularly where a code 2 has been called clinically and the surface called 0 from the photograph (n = 252). Whilst misclassifications are always a possibility i.e. a fissure sealant called as a restoration or vice versa, the data would suggest there might be issues with either examiner thresholding or possible optical confounding issues with the intra-oral images particularly at low caries severity.

**Table 5:** Crosstab data for photographic and clinical ICDAS scores for the upper right first molar (occlusal surface)

		**Photographic ICDAS score UR6 occlusal**							
		**0**	**2**	**3**	**4**	**5**	**6**	**F**	**S**
**Clinical ICDAS Score UR6 Occlusal**	**0**	**506**	8	0	0	0	0	2	5
	**2**	252	**327**	26	4	2	0	7	7
	**3**	2	66	**48**	3	1	0	1	1
	**4**	2	25	15	**25**	4	0	1	1
	**5**	0	1	5	1	**7**	0	2	1
	**6**	0	0	0	0	0	**7**	0	0
	**F**	10	3	0	0	0	0	**138**	4
	**S**	4	1	2	0	0	0	12	**187**

Note: Clinical ICDAS code 1 incorporated into Clinical ICDAS code 2.

**Figure 3:**
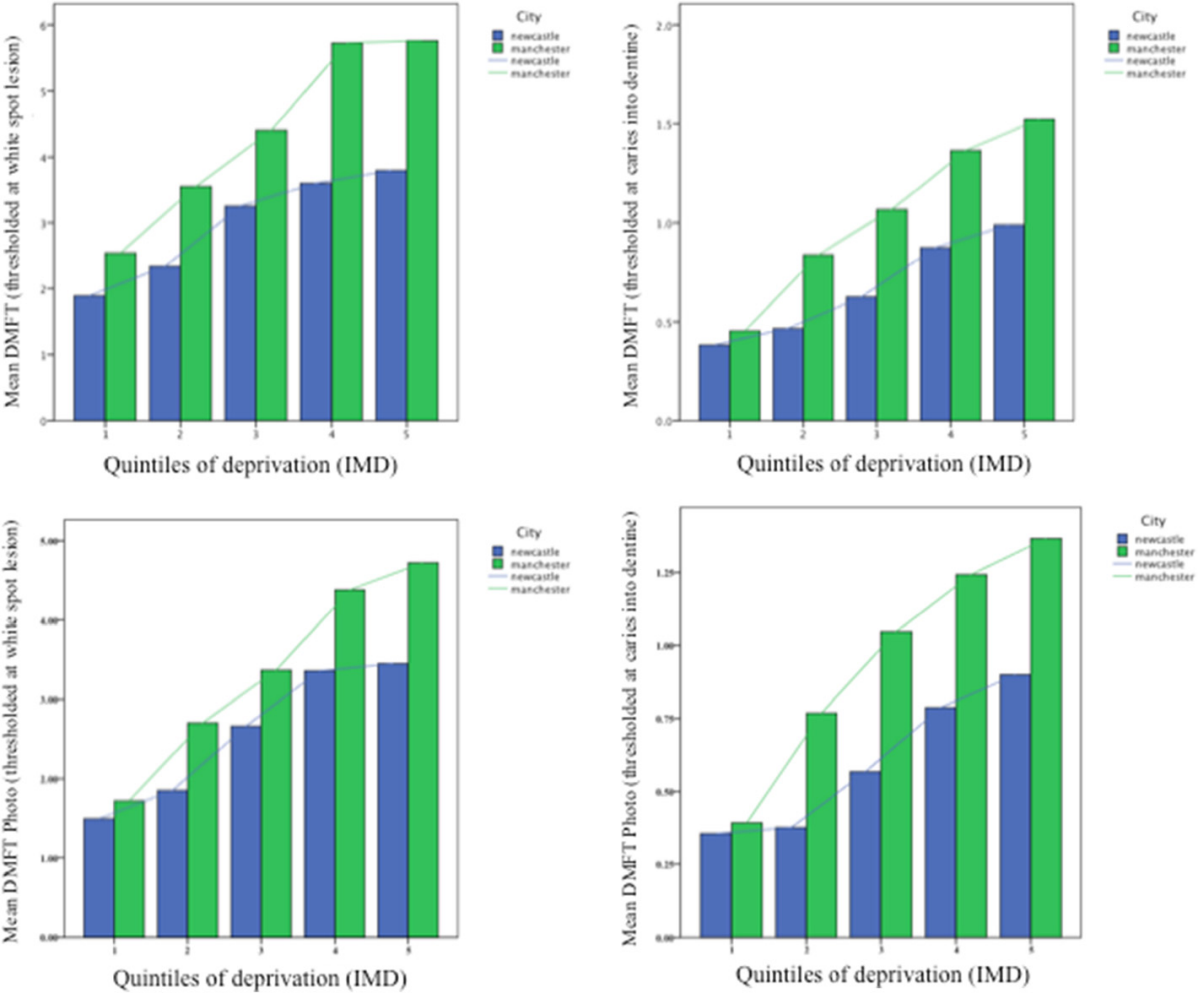
Bar chart of mean DMFT over each quintile of deprivation for each city demonstrating a reduction in social gradient for caries and deprivation in the fluoridated population for both clinical and photographic scores.

The association between quintiles of deprivation and mean DMFT is shown in Table 6, with 1 being the least deprived and 5 being the most deprived. The data demonstrates for both thresholds there was an increase in mean DMFT with increasing deprivation for both the fluoridated and non-fluoridated populations. However, the social gradient between caries and deprivation appeared to be lower in Newcastle when compared to Manchester. This is illustrated in Figure 3. There were significant differences between Newcastle and Manchester across each quintile of deprivation for both white spot lesion threshold and caries into dentine (Mann–Whitney U Test; p < 0.05). The only exception to this was the least deprived quintile, where caries in Newcastle was lower compared to Manchester, but this was not statistically significant for both caries detection thresholds.

**Table 6:** Descriptive data for caries and each quintile of deprivation for white spot lesion and caries into dentine

**Quintile of deprivation**	**Clinical scores**					**Photographic scores**				
	**Newcastle**		**Manchester**		**Mann–Whitney U Test**	**Newcastle**		**Manchester**		**Mann–Whitney U Test**
	**N**	**Mean D**_**1-6**_**MFT white spot lesion (SD)**	**N**	**Mean D**_**1-6**_**MFT white spot lesion (SD)**		**N**	**Mean D**_**1-6**_**MFT white spot lesion (SD)**	**N**	**Mean D**_**1-6**_**MFT white spot lesion (SD)**	
**1**	183	1.89 (2.38)	173	2.54 (2.87)	P < 0.05	183	1.50 (2.27)	173	1.72 (2.21)	P < 0.05
**2**	197	2.34 (2.41)	160	3.56 (3.16)	P < 0.001	197	1.85(2.36)	160	2.71 (2.79)	P < 0.001
**3**	213	3.25 (3.00)	148	4.41 (3.51)	P < 0.05	213	2.67 (2.78)	148	3.37 (2.99)	P < 0.05
**4**	127	3.61 (2.84)	226	5.73 (3.98)	P < 0.001	127	3.36 (3.13)	226	4.38 (3.55)	P < 0.05
**5**	190	3.80 (3.09)	166	5.76 (4.11)	P < 0.001	190	3.45 (3.12)	166	4.72 (3.71)	P < 0.001
**Quintile of deprivation**	**Clinical scores**					**Photographic scores**				
	**Newcastle**		**Manchester**		**Mann–Whitney U Test**	**Newcastle**		**Manchester**		**Mann–Whitney U Test**
	**N**	**Mean D**_**4-6**_**MFT caries into dentine (SD)**	**N**	**Mean D**_**4-6**_**MFT caries into dentine (SD)**		**N**	**Mean D**_**4-6**_**MFT caries into dentine (SD)**	**N**	**Mean D**_**4-6**_**MFT caries into dentine (SD)**	
**1**	183	0.38 (0.86)	173	0.45 (0.88)	n.s.	183	0.36 (0.74)	173	0.39 (0.83)	n.s.
**2**	197	0.47 (1.02)	160	0.84 (1.23)	P < 0.001	197	0.38 (0.87)	160	0.77 (1.14)	P < 0.001
**3**	213	0.62 (1.11)	148	1.07 (1.52)	P < 0.05	213	0.57 (1.03)	148	1.01 (1.40)	P < 0.001
**4**	127	0.87 (1.40)	226	1.37 (1.73)	P < 0.05	127	0.79 (1.43)	226	1.24 (1.61)	P < 0.05
**5**	190	0.99 (1.40)	166	1.52 (1.79)	P < 0.001	190	0.90 (1.28)	166	1.36 (1.42)	P < 0.05

**Figure 4:**
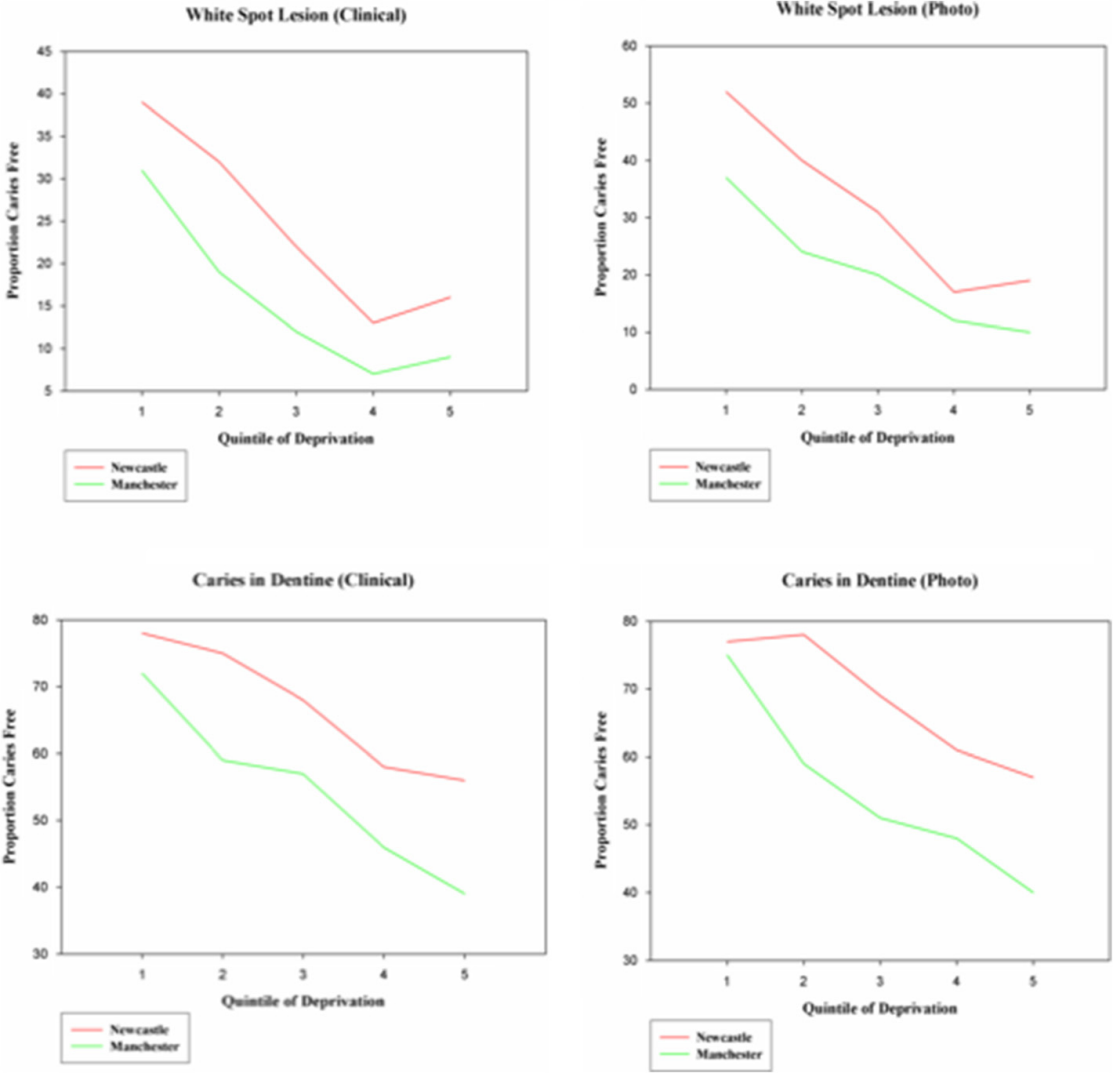
Proportion of “caries free” subjects in each quintile of deprivation for each detection technique and threshold.

Data was generated for the proportion of subjects who were “caries free” in each quintile of deprivation. This was performed for both detection thresholds (white spot lesion and caries into dentine) and both detection methods (clinical and remote photographic scoring). This is illustrated in Table 7. The data demonstrated for each quintile of deprivation and for both detection methods and thresholds there were greater numbers of “caries free” subjects in fluoridated Newcastle compared to non-fluoridated Manchester. Chi Square Tests showed statistically significant differences between Newcastle and Manchester across most of the quintiles of deprivation (Table 7). The differences between Newcastle and Manchester line graphs of this data (Figure 4) demonstrate the differences between the fluoridated and non-fluoridated populations. The difference in gradient between the lines appears to be greater when considering caries into dentine for both clinical and photographic scoring. It would appear in the fluoridated population in Newcastle there is lower gradient between caries and deprivation for caries into dentine compared to Manchester. When considering caries at white spot lesion, the difference in gradient is less pronounced but the proportion “caries free” remains consistently higher in Newcastle.

**Table 7:** Proportion of subjects “caries free” in each quintile of deprivation for each detection method and threshold

		**Proportion “Caries free” for each quintile of deprivation**				
		**1**	**2**	**3**	**4**	**5**
**White spot lesion Clinical**	**Newcastle**	39%	32%	22%	13%	16%
	**Manchester**	31%	19%	12%	7%	9%
	**Chi Square test**	p > 0.05	p <0.01	p < 0.05	p = 0.05	p > 0.05
**Caries in dentine Clinical**	**Newcastle**	78%	75%	68%	58%	56%
	**Manchester**	72%	59%	57%	46%	39%
	**Chi Square test**	p > 0.05	p < 0.01	p < 0.05	p < 0.05	p < 0.01
**White spot lesion Photo**	**Newcastle**	52%	40%	31%	17%	19%
	**Manchester**	37%	24%	20%	12%	10%
	**Chi Square test**	p < 0.05	p < 0.01	p < 0.05	p > 0.05	p < 0.05
**Caries in dentine Photo**	**Newcastle**	77%	78%	69%	61%	57%
	**Manchester**	75%	59%	51%	48%	40%
	**Chi Square test**	p > 0.05	p <0.01	p < 0.05	p < 0.05	p < 0.01

The prevalence and severity of fluorosis (TF > 0) on the maxillary central incisors in Newcastle and Manchester was obtained from the blinded scoring of photographs, the results are described in Table 8. In total there were 1775 subjects with satisfactory photographic information (906 Newcastle; 869 Manchester). The prevalence of fluorosis (TF > 0) in fluoridated Newcastle was 55%; in non-fluoridated Manchester it was 27%. In Newcastle, 48% of subjects had TF scores of 1 or 2 and 7.1% of subjects had TF scores of 3 or greater. In Manchester the corresponding values were 26% and 1.2% respectively. Data from repeat scoring of the photographic images were available for 98 subjects (selected at random). Very good agreement was found between the initial scoring and repeats for scoring at a subject level (weighted Kappa =0.75) [47].

**Table 8:** Descriptive data for fluorosis TF scores

	**City**				**Mann Whitney U**
	**Newcastle fluoridated**		**Manchester non-fluoridated**		
	**Number**	**%**	**Number**	**%**	
**Fluorosis TF Score**					U = 264614, z = −13.025, p < 0.0001
**0**	410	45%	638	73%	
**1**	355	39%	209	24%	
**2**	79	9%	16	2%	
**3**	53	6%	4	1%	
**4**	8	1%	0	0%	
**5**	1	0.1%	2	0.2%	
**Total**	**906**		**869**		

Initial comparisons of the data between Newcastle and Manchester for caries and fluorosis were carried out using chi-square tests and Prevalence Risk Ratios (Table 9). When considering the presence or absence of caries at a threshold of white spot lesion, subjects in Newcastle were 0.88 times as likely to have caries than subjects in Manchester (chi square; p < 0.001). At a threshold of visible caries into dentine, subjects in Newcastle were 0.70 times as likely to have caries than subjects in Manchester (chi square; p < 0.001). Subjects in Newcastle were 2.06 times as likely to have fluorosis than subjects in Manchester (chi square; p < 0.001). When the severity of fluorosis was thresholded at TF 3 or higher this rose to 7.00 times as likely in Newcastle compared to Manchester (chi square; p < 0.001).

**Table 9:** Chi-squared tests and Prevalence Risk Ratios for caries and fluorosis

	**Condition**	**Manchester**	**Newcastle**	**χ**^**2**^**chi square**	**Prevalence risk ratio**
**Clinical Caries: Threshold white spot lesion**	No obvious Caries	133 (15%)	228 (25%)	P < 0.001	Prevalence Risk Ratio for Caries 0.88 in Newcastle
	Obvious Caries	740 (85%)	682 (75%%)		
**Clinical Caries: Threshold caries in dentine**	No obvious Caries	473 (54%%)	614 (68%)	P < 0.001	Prevalence Risk Ratio for Caries 0.70 in Newcastle
	Obvious Caries	400 (46%)	296 (32%)		
**Photo Caries: Threshold white spot lesion**	No obvious Caries	177 (20%)	298 (33%)	P < 0.001	Prevalence Risk Ratio for Caries 0.84 in Newcastle
	Obvious Caries	696 (80%)	612 (67%)		
**Photo Caries: Threshold caries in dentine**	No obvious Caries	475 (54%)	626 (69%)	P < 0.001	Prevalence Risk Ratio for Caries 0.67 in Newcastle
	Obvious Caries	398 (46%)	284 (31%)		
**Fluorosis**	No fluorosis	638 (73%)	410 (45%)	P < 0.001	Prevalence Risk Ratio for Fluorosis 2.06 times as high in Newcastle
	Fluorosis TF 1-5	231 (27%)	496 (55%)		
**Fluorosis**	Fluorosis TF 0-2	863 (99%)	844 (93%)	P < 0.001	Prevalence Risk Ratio for Fluorosis 7.00 times as high in Newcastle
	Fluorosis TF 3-5	6 (1%)	62 (7%)		

The association of age at exam and deprivation with the outcomes of caries and fluorosis were explored using logistic regression models. As a result of the potential loss of information from the photographic scores for caries the analysis comparing the two localities was carried out using the clinical ICDAS caries scores. This is illustrated in Table 10. The explanatory variables of city (fluoridation status), age at exam and quintile of IMD were entered into a logistic regression model with the presence or absence of caries into dentine as the outcome variable. All three variables were statistically significant. The Odds Ratio for developing caries of 1.84 (95% CI 1.50, 2.26) for children in Manchester compared to Newcastle (assuming other explanatory variables held constant). The model also demonstrated increasing Odds Ratios for caries with each increase in quintile of deprivation (but not all increases were statistically significant) and an Odds Ratio of 1.35 (95% CI 1.12, 1.62) for developing caries in to dentine with each additional year of life. The model was shown to have good predictive value (PPV = 56.8% / NPV = 65.7%). The positive predictive value is defined as the proportion of subjects with positive results who are correctly identified and is critically dependent on the prevalence of the condition under investigation.

**Table 10:** Logistic regression models for caries and fluorosis

			**95**% **CI for Odds ratio**		
	**B (SE)**	**Sig**	**Lower**	**Odds ratio**	**Upper**
**Caries into dentine**					
Included					
Constant	−5.041 (1.151)				
City	0.610 (0.104)	P < 0.001	1.500	1.840	2.258
Age at Exam	0.298 (0.093)	P = 0.001	1.123	1.347	1.616
IMD quintile 2	ns	ns	-	-	-
IMD quintile 3	0.496 (0.169)	P = 0.003	1.179	1.642	2.288
IMD quintile 4	0.878 (0.168)	P < 0.001	1.730	2.406	3.345
IMD quintile 5	1.117 (0.166)	P < 0.001	2.05	3.056	4.234
R^2^ =0.088 (Nagelkerke) Model χ^2^ (6) = 119.3, p < 0.0001 Hosmer & Lemeshow chi square = 4.804 sig = .778 PPV = 56.8%; NPV = 65.7%					
**Caries white spot lesion**					
Included					
Constant	−4.511 (1.325)				
City	0.747 (0.122)	p < 0.001	1.622	2.11	2.680
Age at Exam	0.357 (0.107)	p = 0.001	1.160	1.430	1.762
IMD quintile 2	0.473 (0.158)	P = 0.003	1.179	1.607	2.190
IMD quintile 3	0.783 (0.166)	p < 0.001	1.580	2.188	3.028
IMD quintile 4	1.423 (0.193)	p < 0.001	2.847	4.152	6.055
IMD quintile 5	1.487(0.187)	p < 0.001	3.065	4.424	6.387
R^2^ =0.13 (Nagelkerke) Model χ^2^ (6) = 165.47, p < 0.0001 Hosmer & Lemeshow chi square = 11.733 sig = .164 PPV = 75.3%; NPV = 57.0%					
**Fluorosis**					
Included					
Constant	−1.42 (0.132)				
City	1.221 (0.103)	P < 0.001	2.78	3.390	4.152
IMD quintile 1	0.411 (0.160)	P = 0.01	1.101	1.508	2.065
IMD quintile 2	ns	ns	-	-	-
IMD quintile 3	ns	ns	-	-	-
IMD quintile 4	ns	ns	-	-	-
R^2^ = 0.11 (Nagelkerke). Model χ^2^ (7) = 154.95, p < 0.0001 Hosmer & Lemeshow chi square = 7.738 sig = .459 PPV = 56.4%; NPV = 69.5%					
**Fluorosis TF3+**					
Included					
Constant	−4.748 (0.468)				
City	2.344 (0.432)	P < 0.001	4.467	10.424	24.325
IMD quintile 1	ns	ns	-	-	-
IMD quintile 2	ns	ns	-	-	-
IMD quintile 3	ns	ns	-	-	-
IMD quintile 4	ns	ns	-	-	-
R^2^ = 0.11 (Nagelkerke). Model χ^2^ (5) = 57.094, p < 0.0001Hosmer & Lemeshow chi square = 2.936 sig = .938 PPV = 0%; NPV = 96.0%					

The model created for caries at a white spot lesion threshold provided an Odds ratio of 2.11 (95% CI 1.62, 2.68) for children in Manchester compared to Newcastle. Once again, Odds Ratios increased as quintile of deprivation increased, as did the Odds Ratio for age at exam. Again, the model was shown to have good predictive value (PPV = 75.3% / NPV = 57.0%). The predictive value for this model appears to be better than the model for caries into dentine. However, this is probably influenced by the increased prevalence when considering caries at a white spot lesion threshold.

Explanatory variables for city (fluoridation status) and quintile of IMD were entered into a logistic regression model with the presence or absence of any fluorosis as the dependent variable. The Odds Ratio for developing fluorosis was 3.39 (95% CI 2.78, 4.15) times greater in Newcastle when compared to Manchester. The effect of deprivation on fluorosis was only significant for subjects in the least deprived quintile of IMD. The Odds Ratio of developing fluorosis was 1.51 (95% CI 1.10, 2.07) for those in the least deprived quintile of IMD when compared to the most deprived quintile. This model was shown to have good predictive value (PPV = 56.4% / NPV = 69.5%).

A logistic regression model looking at the presence or absence of fluorosis at a severity of TF Index of 3 or higher produced an Odds Ratio of 10.42 for Newcastle compared to Manchester. However, whilst this was significant, the model was deemed to be unstable because of the low numbers of cases in at least one of the cells. Caution should be taken when interpreting the results from this model.

Each model was tested for interactions between the variables. It was found there were no significant interactions in any of the models and the data presented is a refit of the models without the tests for interactions.

## Discussion

The results support the existing evidence from other studies conducted in the UK that water fluoridation reduces inequalities in health by reducing the social gradient between deprivation and dental caries [24,29-32]. Using IMD as a measure of deprivation enabled a more accurate assessment of deprivation for individuals by allocation of a score for at a LSOA level via postcode rather than at the electoral ward level. This avoided analysis of the data by mean DMFT scores at a ward level. By initially selecting schools through %FSME, it facilitated a more balanced profile of deprivation albeit resulting in selected populations. However, this study demonstrated the benefits and trade offs associated with the use of fluorides in dentistry remain an important consideration. The decision to report on DMFT rather than DMFS was based on a number of factors; water fluoridation is a broad population based intervention and as such DMFT was deemed to be a more appropriate reporting tool. It avoided any potential issues surrounding the M (missing) component at a surface level encountered using DMFS and it was consistent with the reporting used for existing national surveys in England.

Despite the significant difference in caries prevalence and severity in Newcastle compared to Manchester, it has been achieved with an increased prevalence in mostly mild fluorosis. The overall prevalence of fluorosis in Newcastle is comparable to that observed by Tabari and Ellwood et al. [48] in a study conducted in Newcastle and non-fluoridated Northumberland but the prevalence of fluorosis at a severity of TF 3 or greater appears to have increased from 3.4% to 7.0% in the ten years separating the two studies. Caution should be taken when interpreting these results. Fluorosis recorded at a level of TF3 is still considered to be mild or mild to moderate on a scale of 0 to 9. The results do suggest there has been an increase in the proportion of individuals within this higher category. However, the severity of the presentation should not be considered as severe fluorosis. While both studies adopted the same index and method of remote scoring of standardized photographs, the primary analysis of the earlier Tabari study employed the use of clinical scores, whereas this study used standardized images, which were magnified. This could potentially result in changes in detection thresholds between the two studies. Furthermore, without the re-scoring of the images from the first study by the examiner of the current study it is not possible to ascertain if personal thresholding or the effect of image magnification in the current study has affected the outcome [49]. As the numbers of subjects in the more severe categories are relatively low, small changes in prevalence of subjects in these higher TF categories may dramatically affect subsequent Odds Ratio calculations.

It is important to remember this study has only reported fluorosis prevalence and severity on the maxillary central incisors. This is largely owing to the fact these teeth are the most practical from which to obtain good images and are considered important in assessing aesthetics. The risk period for fluorosis for these teeth is open to debate, but it is generally accepted they are generally at greatest risk from birth up to the age of three years [50-52]. In essence this study is examining the effects of fluoride intake in the early years of life. However, risk assessments for fluorosis should not be confined to the maxillary central incisors but to the whole dentition taking into account the overall intake of fluoride in terms of dose and length of duration of exposure [51]. It was not practical to assess fluorosis on the remaining dentition therefore it is not possible to draw conclusions on any differences in fluorosis prevalence and severity outside of the parameters defined in this study. Fluoride intake from feeding practices during growth and development and oral hygiene practices may have an effect on fluorosis presentation on teeth erupting after the maxillary central incisors. It is entirely plausible the apparent increase in fluorosis prevalence at higher severities in Newcastle is as a result of greater fluoride intake derived from an additive effect of water fluoridation and potential misuse of fluoridated dental products. This is not a novel concept [53,54] and has been addressed in some areas with fluoridated water supplies. The Republic of Ireland has recently reduced the recommended content of fluoride in water supplies in 2007 from 1ppmF to 0.7ppmF following a review [55] and in the United States the U.S. Health and Human Services together with the Environmental Protection Agency has recommended a similar reduction in water fluoride content following a report from the National Academies of Science [56]. This will require evaluation to monitor not only changes in fluorosis prevalence and severity but also any detrimental effects on caries prevalence particularly in more deprived communities.

This study supports the existing evidence showing the use of water fluoridation and fluoridated dentifrice has a greater impact on caries levels than the use of fluoridated dentifrice alone. Studies in the permanent dentition have provided variable results and it was suggested by Ellwood and O’Mullane [29] that it is more difficult to demonstrate differences when population caries levels are low. When examining the confidence intervals for mean DMFT for Newcastle and Manchester at both thresholds of detection there is no overlap suggesting significant differences exist in caries levels. The use of ICDAS criteria in calculating DMFT permits analysis of early carious lesions as well as the more traditional visible caries into dentine employed by the NHS DEP. The ICDAS index is a potentially useful epidemiological tool as it could facilitate the longitudinal monitoring of early carious lesions and explore the progression/regression of such lesions in an individual over time. Examination of the results of this study reveal the difference in prevalence between the fluoridated population and the non-fluoridated are reduced when the caries is reported at a threshold of white spot lesions. The question is raised whether water fluoridation prevents or merely delays the progression of early caries. This could only be answered by longitudinal examination but the findings of this study are consistent with those conducted in the Netherlands in Tiel and Culemborg where differences were found between fluoridated and non-fluoridated populations in the prevalence of caries (including early “white spot” lesions) although it should be stressed there was no assessment of lesion activity undertaken in this study.

The logistic regression models for caries demonstrated good levels of prediction (based upon the positive and negative predictive values) when considering fluoridation status, deprivation and age at examination as explanatory variables. However, the effect size was relatively low suggesting other factors influenced caries risk to a greater extent. It is obvious both diet and oral hygiene practices will have a great effect on caries risk for an individual and are important considerations to include in the development of caries risk models to improve on current models lacking valid and reliable means of accurately predicting caries risk [57]. Nevertheless it has been demonstrated that deprivation and fluoridation status will have an effect on caries risk and are important considerations to make when evaluating both passive population based preventative interventions such as water fluoridation and targeted interventions such as topical fluoride applications for high caries risk individuals.

There were several logistical difficulties encountered during this study. All of the subjects were examined during academic term time in a school setting, which created several logistic difficulties during both the planning and execution phases. Secondary schools have a congested curriculum and required the permission not only of the local authorities and the head teachers of each school to facilitate time and space to minimize disruption to the academic timetable but also physical space in which to perform the examinations. Whilst this was generally successful in enabling examinations there were a number of instances where conflicts in school timetables could not permit additional visits to examine absentees or pupils with alternate commitments. This was reflected in Manchester where there were a disproportionate number of subjects unavailable owing to proximity to the Christmas holidays and related events organized in school (Figure 1).

Additional difficulties and limitations should be considered in a study of this nature. Following the recommendations from the York Report [36] it is accepted that a cross sectional study is not the most robust design for assessing the impact of water fluoridation. However, the cost implications for a study design that would include prospective monitoring of birth cohorts, serial cross sectional surveys that include analysis of diet and total fluoride intake with anthropometric measurements would be cost prohibitive and beyond the scope of this project. Nevertheless, the aforementioned are important considerations to be taken during study design.

This study did include an assessment of dietary intake of sugars through an interview process with a dietician on a representative cohort, but this was not a practical consideration for the entire study population and acted merely to demonstrate there were no significant differences between the populations with respect to caries risk from dietary intake of NMES. The oral hygiene practices questionnaire was unable to assess previous fluoride intake and any interview recall of infant practices would be prone to bias. Assumptions were made that most subjects (if not all) used fluoridated dentifrice and they were questioned on use of fluoride supplements that only elicited a positive response by very few subjects (Figure 1). The results are interesting to report whereby significantly more subjects in Manchester reported not rinsing after brushing which would assist in the maintenance of the oral fluoride reservoir. However, it is important to note when considering the study population as a whole an overwhelming proportion are not following the current recommendations of expectorating but not rinsing after brushing [58]. During the study design phase the option of including anthropometric measurements was discussed but as it would have generated little additional value in context with other captured data and potentially impacted upon consent rates, it was decided not to pursue this option.

The consent rate is an important consideration to make in a study of this nature with respect to the validity of the data and the representative value of the study population. The consent rate when considering all subjects available for examination was 63.1% (64.3% Newcastle; 61.7% Manchester). These figures are low when considering the level of consent rates expected for observational surveys, but in the absence of a negative consent process the consent rates in this study are commensurate with those of a survey using a positive consent process such as the NHS DEP. The demographics and caries status of the subjects who did not participate remain unknown, as is the impact their data would have on study outcomes. There is the possibility this would have the effect of underestimating the effect of deprivation and caries, as it would be reasonable to assume subjects that did not provide consent or attend for examination had high caries levels.

The populations examined in this study should be correctly described as being selected populations. Whilst most of the state secondary schools in Newcastle participated in the study there were three state schools that did not participate. Public and private schools were not approached and it would be assumed the pupils from these schools would reside in more affluent areas. In order to minimize bias between the populations, the schools approached in Manchester were targeted to enable an equitable balance in deprivation. Therefore many of the inner city schools in Manchester were not approached owing to either high non-lifetime residency of pupils or the%FSME profile did not match an equivalent school in Newcastle.

The results from the use of the intra-oral camera for remote scoring demonstrated a potential means of blinded assessment. It had been hypothesised that the use of the camera would reduce the level of potential examiner bias and the images would be able to facilitate longitudinal assessment through the use of video repositioning (VidRep) software. The DMFT for the photographs were consistently lower than the clinical scores and it was felt that the lack of clear visualization of the interproximal surfaces together with confounding from specula reflection might have impacted on the results. However, the technique demonstrated the ability to discriminate between the populations and comparison of the ICDAS scores for the occlusal surfaces of the first molars between the photograph and clinical scores produced a weighted Kappa statistic of 0.83 suggesting a very good level of agreement between the methodologies when comparing the same high caries risk surface [47]. The similarities between the clinical and photographic scoring methods are encouraging despite the acknowledged confounding issues. Additional work is required to improve intra-oral image capture and investigate the reasons for the differences in severity scores but the incorporation of a polarizing filter may reduce the effect of specula reflection on subsequent image scoring. The difficulties associated with obtaining suitable images to visualize the interproximal surfaces may be more problematic to address.

Drawing any conclusions from the results of this study and those of the NHS DEP (which were carried out in largely the same population) are interesting but do require caution and qualification. The aim of this study was to utilize the ICDAS criteria in order to detect early caries rather than at the D_3_ level used in the BASCD criteria employed in the NHS DEP survey. Comparisons between indices and the pragmatic use of ICDAS with single representative scores on surfaces have been reported in the literature with favourable outcomes [59-61]. The differences between the data for this study and the NHS DEP survey for caries into dentine (ICDAS code 4 and D_3_) and also for lifetime versus non lifetime residents in Newcastle are interesting and would require a more thorough investigation to validate but the inference from the data is there is a possible effect on caries for lifetime residents in the fluoridated population examined in this study compared to the population examined in the NHS DEP.

## Conclusions

The results of this study support existing work suggesting water fluoridation together with the use of fluoridated dentifrice provides improved caries prevention over the use of fluoridated dentifrice alone. The social gradient between caries and deprivation appears to be lower in the fluoridated population compared to the non-fluoridated population, particularly when considering caries into dentine, demonstrating a reduction in inequalities of oral health for the most deprived individuals in the population. However, the risk of developing mostly mild fluorosis is increased in the fluoridated population when associated with the widespread use of fluoridated dentifrice, particularly in the least deprived individuals. The use of ICDAS may provide greater flexibility to report and monitor early carious lesions more favourably than existing methods employed in oral health surveys. The use of intra-oral cameras for blinded caries scoring demonstrated the ability to discriminate between a fluoridated and non-fluoridated population and has good potential for blinded caries assessment but the technique requires additional work to address potential information loss and confounding issues.

## Abbreviations

BASCD: British Association for the Study of Community Dentistry; %FSME: percentage free school meal entitlement; CI: confidence interval; DEP: Dental Epidemiology Programme; DMFT: Decayed Missing and Filled Teeth; D_1-6_MFT: DMFT from ICDAS codes threshold at white spot lesion; D_4-6_MFT: DMFT from ICDAS codes threshold at caries into dentine; ICDAS: International Caries Detection and Assessment System; IMD: Index of Multiple Deprivation; LSOA: Lower Super Output Area; NHS: National Health Service; NMES: Non-milk extrinsic sugars; NPV: Negative predictive value; PPV: Positive predictive value; SPSS: Statistical Package for Social Sciences; TF: Thylstrup & Fejerskov Index; VidRep: Video Reproducibility.

## Competing interests

None of the authors are aware of any competing interests in the production of this manuscript.

## Authors’ contributions

MGM prepared the protocol, conducted the fieldwork, was involved in the analysis of data and wrote the manuscript. RPE provided input into the study design and the manuscript. AM acted as local Investigator and provided input into study design and he manuscript. NB co-ordinated the study, conducted fieldwork and participant instruction on questionnaire completion. MG conducted the statistical analysis. IAP was Principal Investigator and provided input into study design and manuscript. All authors read and approved the final manuscript.

## Pre-publication history

The pre-publication history for this paper can be accessed here:

http://www.biomedcentral.com/1471-2458/12/1122/prepub
